# How does cognition determine an individual’s fitness? A systematic review of the links between cognition, behaviour and fitness in non-human animals

**DOI:** 10.1098/rstb.2024.0118

**Published:** 2025-06-26

**Authors:** Joah Robert Madden, Samuel Ellis, Luc Lens, Frederick Verbruggen

**Affiliations:** ^1^ Department of Psychology, University of Exeter, Exeter EX4 4QG, UK; ^2^ Centre for Research in Animal Behaviour, University of Exeter, Exeter EX4 4QG, UK; ^3^ Department of Biology, Ghent University, Ghent 9000, Belgium; ^4^ Department of Experimental Psychology, Ghent University, Ghent 9000, Belgium

**Keywords:** cognition, fitness, cognitive ecology, survival, reproductive output, cognitive performance

## Abstract

Recent efforts to understand the evolution of cognition have adopted a within-species approach where cognitive performance, and its effects on behaviour, is correlated with fitness benefits and, if related, is assumed to be selected for. We reviewed 45 studies taking this approach, involving 26 species and describing 211 relationships between behavioural measures of cognition and fitness, to explore broader patterns underlying the evolution of cognition. First, we explored patterns in the strength and direction of selection. We found generally weak support for a relationship between cognition and fitness, with >70% of raw published relationships being statistically non-significant, with an even smaller likelihood once co-variates were accounted for. Where significant relationships were found, they were predominantly, but not exclusively, positive, with individuals exhibiting faster learning or more accurate memory also displaying greater (proxy) fitness. Second, we tested how selection might act under different circumstances. A relationship with fitness was more likely when general, rather than specific cognitive entities were considered, and when the fitness measure corresponded to survival rather than reproductive output. Consequently, the study of within-species cognitive evolution remains in its early stages, with evidence that is both incomplete and inconclusive. However, it potentially offers a powerful opportunity to explore the structure of cognition, trade-offs, constraints and the way that it links to behaviours.

This article is part of the Theo Murphy meeting issue ‘Selection shapes diverse animal minds’.

## 1. Introduction

To understand how Cognition evolves, two complementary approaches have been taken. The comparative approach (Comparative Cognition) compares different species (or populations) and investigates how they differ in whether they possess particular Cognitive Entities, or in the Cognitive Performances they demonstrate, and then searches for ecological, life-history or morphological features that correspond to these differences while accounting for their patterns of relatedness [1]. A second, more recent approach adopts the Darwinian method [2–4], which looks within species, considering differences in the possession of cognitive entities or expression of cognitive performance between individuals [5] and the fitness consequences in natural environments that arise from these differences. This approach, known as Cognitive Ecology [6], allows researchers to understand how selection may act on the cognitive entity of interest and thus how it may have evolved [7]. This approach may be experimental, with deliberate artificial selection on captive populations, intentionally mimicking putative natural selective forces, for particular cognitive entities or levels of cognitive performance over multiple generations (e.g. [8]). Alternatively, researchers may take an observational approach, in which they assay the cognitive entities and/or performances of large numbers of individuals, often in the wild (e.g. [4,9,10]). They then follow their fates, correlating these to the presence of a cognitive entity or expression of their cognitive performance earlier in life and inferring causal links between these and an individual’s (proxy) fitness outcome, and thus illuminating how selection may act to shape the presence or extent of the cognitive entity.

The Cognitive Ecology approach is based on the same principles as the Behavioural Ecology approach, a powerful methodology developed in the 1970s that focuses on understanding the adaptive significance of behaviours in ecological contexts [11]. Instead of focusing on how a particular behaviour affects an individual’s fitness, researchers of the evolution of cognition assume that one or more cognitive entities underlie the behaviour. The expression of the behaviour depends on the performance of these underlying entities. Consequently, when selection acts on the behaviour itself, the underlying cognitive entities and their performance levels are assumed to co-evolve over time. For example, food-caching efficiency and retrieval can be observed and are assumed to be underpinned by cognitive entities including spatial memory and inhibitory control [12]. Individuals are seen to differ in caching and retrieval performance, presumably because of differential performance in the underlying cognitive entities, and this is expected to influence over-winter survival. Differences in survival exert a selective pressure on cache behaviour, and this in turn is inferred to act on the cognitive performances and entities underpinning it (e.g. [13]). This progression, from cognition to behaviour to fitness outcome, has been the basis of a suite of recent studies. In these studies, the link between cognitive performance and (proxy) fitness outcomes has been explored by assaying the cognitive entities that are presumed to underpin the behaviours of interest instead of, or in addition to, measuring the behaviours themselves (e.g. [4,14,15]). The presence of a cognitive entity or degree of cognitive performance is assessed in each individual using Psychometric Tests, administered in a uniform manner across individuals, permitting the detection of cognitive entities or measuring performance, with attempts to control for potential confounds such as opportunity, prior experience and/or motivation [5].

Studies of Cognitive Ecology vary in the Cognitive Level that they focus on. Some consider specific, psychologically defined cognitive entities that may operate in a rather narrow range of particular contexts (e.g. short-term spatial memory [16], speed of learning shape discrimination [17]), which we term Specific Entities. Other studies consider broader cognitive entities that might be composed of suites of specific entities to produce what we term Broad Entities (e.g. problem Solving [14], innovation [18]) which may operate in a wide range of behavioural contexts. Still other studies consider composite measures presumed to provide an overall measure of cognition that is determinant in all of the animal’s behaviours, perhaps analogous to ‘*g*’ (e.g. [19]) derived from an integration of multiple measures of specific and broad entities to produce a single component (e.g. [15,20]), which we term General Entities. Thus, as cognitive level increases, such studies become less specific about the particular cognitive entity of interest and the resulting behaviour.

Defining and measuring Fitness is also complex [21], especially for free-living long-lived species. Consequently, researchers may use Proxy Fitness Measures, which become easier to collect as they become less directly linked to fitness, but obviously the confidence that we might have in how strongly they might be subject to selection also decreases. Despite the complexity of data collection, in terms of both cognitive testing and life-history tracking, there are now a sufficient number of published studies to permit a preliminary evaluation of overall patterns and to compare and review what they can tell us about how cognition may evolve through selection.

To address this broader topic, we can ask two key questions about the evolution of cognition through natural selection. First, how do patterns in the strength and direction of selection affect cognitive traits and their evolution? Second, how might selection vary under different circumstances, such as differences in cognitive level or life-history context? For our first question, in the case of cognitive entities and their performance, it is often considered that there is directional selection favouring continued exaggeration: individuals that might learn faster, remember more information or exert faster or stronger executive control might be fitter. Typically, humans might crudely describe such individuals as being Smarter, and intuitively selection might be expected to favour these individuals. However, as with other non-cognitive traits, selection is usually tempered by costs. The neural architecture and processing required for additional or exaggerated cognitive entities and performance is expensive, taking space and energy (e.g. [22,23]), and cognitive entities and performances may conflict with one another, leading to trade-offs (e.g. [24,25]). There may also be circumstances where it is not beneficial to rapidly learn new behaviours or remember ever more information (e.g. [26,27]). Therefore, although we expect to find both positive and negative relationships between an individual’s cognitive performance and its fitness, it is unknown whether one is more likely than the other. Detecting these relationships may be difficult because, as with other studies attempting to explain evolutionary trajectories from relationships seen between traits and fitness at a single timepoint, beneficial traits have already reached fixation, so that no variation currently exists and no positive or negative relationships can be observed (e.g. [28]).

For our second question, we can test how selection might act differently under different circumstances. The strength and direction of any relationships between cognition and fitness may depend on the cognitive level being considered and/or the life-history context that cognition is deployed in. More specific cognitive entities, tied to a limited range of behaviours, may experience more consistent selection, making fitness associations easier to detect. Broad or general entities are likely to contribute to a wider range of behaviours and thus be subject to a range of differing selection pressures. These might all individually drive exaggeration in the same direction, in which case it might be more likely that relations with fitness are reported. Alternatively, selection may act differently on each behaviour that the broad entities contribute to, eroding the strength of selection on any particular cognitive entity, in which case it might be less likely that relations are reported as the cognitive level becomes more general. In addition to the cognitive level, we might expect to find differences in the likelihood and direction of relationships depending on the fitness measure assessed. For example, reproductive fitness is typically only available to adults, which have lived longer and thus had more opportunity to benefit from learning behaviours and acquiring and storing information. In contrast, survival may be highly dependent on events (predation, sudden environmental changes) that are encountered rarely and often in early life, offering little opportunity for learning or memory to operate. Survival may also depend on the ability to respond quickly to immediate threats, which may involve more instinctive or less cognitively demanding behaviour. Therefore, we might expect to find different relationships, indicative of differential selection pressures, between cognition and measures of reproductive fitness compared with those with (proxy) measures of survival fitness.

## 2. Methods

### (a) Literature review

**Figure 1.**
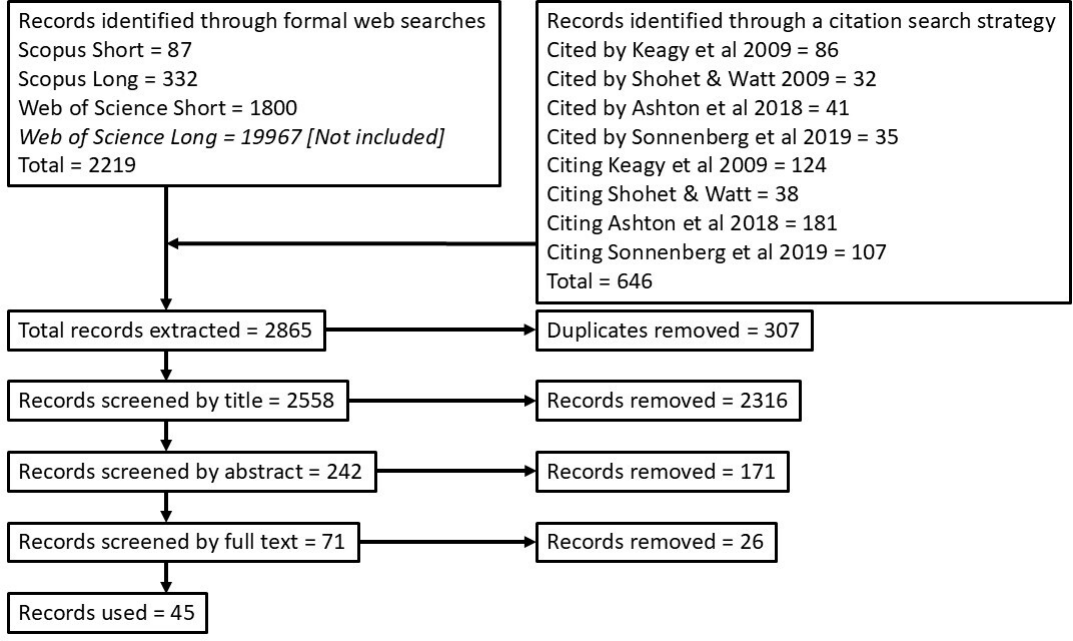
Summary of literature search and selection of studies for use in this review.

We searched for studies that measured the cognitive performance of many individuals in one or several cognitive entities, using abstract psychometric tasks, and then followed the fates of those individuals, reporting (proxy) measures of their fitness so that authors could infer selective pressures on cognition. The searches were conducted on 12−14 August 2024. We initially searched Scopus using the prompt (('cognitive AND ability' OR 'cognitive AND performance') AND (fitness OR 'reproductive AND success' OR 'survival')) AND NOT (human). This produced 87 results. We extended the Scopus search to (cogniti* AND (fitness OR 'reproductive AND success' OR 'survival')) AND NOT (human). This produced 332 results. We then search Web of Science using the Scopus short prompt (('cognitive ability' OR 'cognitive performance') AND (fitness OR ‘reproductive success’ OR ‘survival’)) NOT (human). This produced 1800 results. We again extended the Web of Science search to (cogniti* AND (fitness OR ‘reproductive success’ OR ‘survival’)) NOT (human). This produced 19 967 results, which was excessive for screening, so we pooled the first three searches to leave us 2219 papers to screen. An initial scan of these papers revealed that several papers that we knew to be appropriate had not been included. We attempted to refine search terms to trade off sensitivity and specificity but could not find search term combinations that captured all papers that we knew *a priori* should be included. Therefore, we complemented our literature search with a ‘citation search’ strategy [29] in which we selected a series of key, well cited papers in the field—two from the earliest period when we were aware that this field of research started, in 2009, and two a decade later [13–15,30], and searched those citing them (*n* = 450) and which they had cited (*n* = 194). We added these to the papers from the literature search and then conducted our screening on 2865 papers. All papers encountered are listed in electronic supplementary material, S3. Our search results are summarized according to PRISMA guidelines [31] in figure 1.

We included papers that measured a specific, named cognitive entity, using an abstract psychometric test, in multiple individuals and that provided (proxy) fitness measures such as behaviours that the authors described as being beneficial or subject to selection. From this set of included studies, we then excluded studies that: were based on brain size rather than cognitive performance (e.g. [32]); were based on populations deliberately selected for brain size and/or cognitive performance (e.g. [33]); used colony-level cognitive or fitness measures; used retrospective cohort studies, e.g. comparing ratios of adults to juveniles as a proxy for survival (e.g. [27]); used highly specific biologically relevant stimuli, e.g. learning about predator identities (e.g. [34]); or proposed that fitness measures caused or predicted cognitive performance (e.g. [35]). Crucially, we only included studies where cognitive performance was tested first and then a (proxy) fitness measure was taken, such that the authors hypothesized that cognition affected fitness.

This search resulted in data from 45 studies, involving 26 species, and produced 211 relationships between measures of cognitive entities and fitness (table 1).

**Table 1.** Summary of the number of species, studies and relationships considered in this review. Full data on studies used are in electronic supplementary material, S2.

	number of species	number of studies	number of relationships
invertebrates	1	3	8
fish	4	6	14
reptiles	1	1	1
birds	16	28	153
mammals	5	7	35

From these studies, we used the terminology for the cognitive entity being measured as given by the authors, even if there was ambiguity. For example, a detour task might be intended by the authors to indicate inhibitory control, yet it may also provide a measure of an individual’s response learning or other factors [36,37]. If the authors consider it to indicate inhibitory control, we also accepted that the measures indicated behavioural inhibition. We also recorded if the relationships were subject to any limitations, e.g. they applied to one sex or age class only, were condition-dependent or were involved in interactions with non-cognitive factors, e.g. body mass (full details of all limitations for each study are given in electronic supplementary material, S2). This was conveyed in a binary yes/no presence of limitations.

We accepted the measures and definitions of the authors of their (proxy) fitness measures. We crudely classified (proxy) fitness as being achieved through reproduction or survival. For example, numbers of fledglings produced, attractiveness of male trait, or numbers of copulations may indicate reproductive output, whereas those relating to risk-taking, foraging efficiency or time foraging may indicate survival. In studies where multiple measures of fitness were presented, we used those that were more likely to be directly related to fitness (e.g. probability of surviving to the next year or number of days survived post-testing measures instead of any more tenuous proxies such as body mass or foraging success).

### (b) Analytical approaches

We initially intended to conduct a formal meta-analysis of the results presented in the papers that we reviewed. However, once we had collated the work, read the results in detail and extracted, where possible, effects and sample sizes, we became unconvinced that a meta-analysis would provide robust conclusions (see electronic supplementary material, S1). Therefore, we opted for a vote-counting approach [38] to explore the direction and distribution of relationships between cognitive performance and fitness. This approach involved counting the number of published relationships presented by the authors as being statistically significant or not, while acknowledging that it may include studies with effect sizes that are not theoretically or biologically meaningful. We classified these relationships as: positive, e.g. faster-learning individuals or those making fewer mistakes survive longer (a.k.a. ‘smarter is better’); or negative, e.g. slower-learning individuals or those making more mistakes survive longer (a.k.a. ‘dumber is better’). Relationships that were not considered to be statistically significant by authors, including those discarded when *α*-values were adjusted for multiple testing, were classified as neutral. We did not conduct power analyses to assess the robustness of these published non-significant relationships.

We analysed the results of a literature survey with two Bayesian linear models. The first model—which we term the Any Effects Model—coded whether a particular tested relationship found a significant relationship as a binary 0/1 output. We consider this Any Effect term to be drawn from a Bernoulli distribution, with probability predicted by the logit of a linear regression with the predictors: cognitive level (domain-specific/domain-general/general intelligence), fitness measure (survival/reproduction), presence of a limitation (0/1; see above); study sample size (logged) and with study identity as a random intercept. The cognitive level and fitness measure terms were allowed to interact such that each cognitive level : fitness measure pair can have a different relationship with the probability of finding an effect. Our second model—the Effect Direction Model—separated the Any Effect term into positive and negative effects. In this model, the outcome (no effect/positive effect/negative effect) was drawn from a multinomial distribution, with the (logit) probability of a specific test falling into a particular category given by the same linear regression as for the Any Effects model. All parameters have weakly informative priors. Models were fitted in Stan via the brms package [39] in R [40]—using Hamilton Monte Carlo chains. Models were checked for model convergence and divergent transitions using prior and posterior predictive checks. We use these fitted models to answer our research questions. For each model we took 4000 draws from the posterior distribution and use this to calculate estimated effects under different parameter combinations: for example, the predicted probability (sometimes referred to as an estimated marginal mean) of finding any effect in a test of a broad entity, using a survival fitness measure, with no limitations and a sample size of 40. The output of this process is a distribution of 4000 predicted probabilities of finding any effect for a given parameter combination. We describe these distributions via their 50% credible interval (which can be interpreted as a 50% probability that the true value lies within this range), the posterior mean and the 95% credible interval. A strength of this approach is that for research questions that require a contrast between two sets of parameters we can directly compare the predicted probabilities at each draw in the posterior and therefore directly infer the probability that one parameter combination, for example, has a higher probability of finding an effect, or that the parameter combination is larger than 0. For these contrasts, we report the probability *P* for a given inequality shown in brackets. For example, in the Any Effects model, *P*(*β*_log(SAMPLE_SIZE)_ *>* 0) can be interpreted as the probability that the log(sample size) parameter is greater than 0 and that there is a positive relationship between sample size and the probability of finding an effect. In all contrasts, if a given variable is not included in the contrast, the effect is either averaged over all values of that level to get the overall effect given an unknown factor level, or for sample size the mean of the raw data is taken.

We are aware that owing to phylogenetic constraints, data from different species may not be independent of one another. However, the phylogeny that contains all our study species was extremely sparse, spanning from insects to mammals, but containing data from only 37 species, with particular clustering of passerines. To explore whether this might distort our findings, we ran models where we included species as the random effect rather than study identity, and we ran models where we included crude taxa (birds, mammals, fishes, reptiles, invertebrates). These model variants were qualitatively (and generally quantitatively) very similar to our reported models (full results from these models is given in electronic supplementary material, S4). The only difference was a decrease in the chance that a positive relationship was greater than a negative relationship (electronic supplementary material, table S4.2 and figure S4.2), and this was probably driven by the over-inclusion of bird studies, for which positive relationships were more likely than negative relationships (electronic supplementary material, figure S4.3). We are also aware that the animals included differed in the conditions that they were tested under and under which (proxy) fitness was assessed. However, definitions of whether study animals were wild or captive were complex. In some studies, wild animals were caught and tested in captivity (e.g. [41]). In others, animals were reared and tested in captivity, but released and subject to fitness consequences in the wild (e.g. [26]). In others, animals were assessed for their cognitive performance and fitness (proxies) entirely in captivity (e.g. 42), while for others, animals were entirely free-living (e.g. [43]). But even for some of these ‘wild’ populations, there was obviously a high degree of habituation/training/human interference, making their status hard to discern (e.g. [15]). Given our relatively small sample size (45 studies) and the likelihood of collinearity with species (see above), we did not include testing and living locations as further variables.

## 3. Results

### (a) Are there any overall patterns of relationships?

Most (149/211, 71%) of the reported relationships between cognitive performance and fitness were neutral. Of the 62 significant relationships, 48 (77%) were positive, with higher (proxy) fitness for individuals that learned associations faster, made fewer errors or exhibited inhibitory control more strongly, and 14 (23%) were negative, with higher (proxy) fitness for individuals that learned associations slower, made more errors or exhibited inhibitory control less strongly. Of the analyses that yielded significant relationships 43/62 (69%) were subject to some limitation (e.g. they applied to one sex or age class only, were condition-dependent or were involved in interactions with another non-cognitive factor, e.g. body mass), similar to those that yielded neutral relationships (99/149, 66%). Most (147/211, 70%) of the published relationships considered (proxy) fitness measures relating to reproductive success, with the remaining 30% considering survival. There were differences in the number of studies of different cognitive levels. Measures of specific entities were the most common (147/211, 70%), with measures of broad entities composing 22% (47/211) and those of general entities making up the final 8% (17/211).

### (b) Patterns of relationships when considering if any significant effect was reported

**Figure 2.**
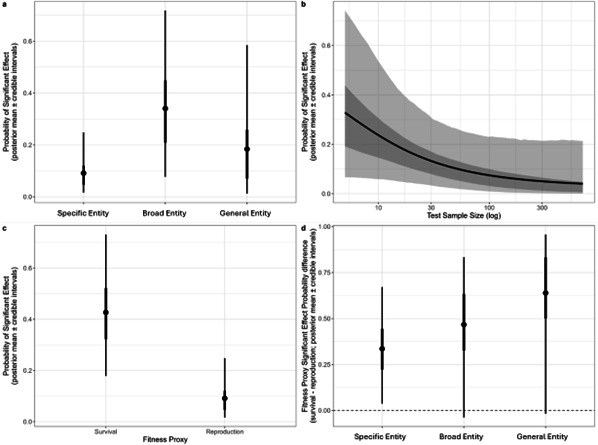
Probability of a test finding a significant effect derived from the fitted Any Effects model. (a–c) The predicted probability of a test reporting a significant effect given (a) the cognitive level tested, (b) the sample size, and (c) the fitness proxy used. (d) The difference in predicted probability of reporting an effect at each cognitive level tested for tests using survival versus reproduction fitness proxies. Predicted differences above 0 indicate that the probability of a study reporting a significant effect is higher when survival rather than reproduction is used as the fitness proxy. In each panel, points/lines show the posterior mean, thicker error bars/darker ribbons show the 50% credible interval, and thinner lines/lighter ribbons show the 95% credible interval.

Overall, the probability of a given test chosen at random from the literature reporting a significant effect is 0.07–0.29 (50% cred. int; post. mean = 0.09, 95% cred. int. = 0.02–0.25). The probability of a significant effect being reported declines with an increase in sample size (*P*(*β*_log(Sample size)_ > 0) = 0.045): for every order of magnitude change in sample size there is a 36–93% (50% cred. int; post. mean = −0.67, 95% cred. int. = −0.10 to −1.54; figure 2b) drop in the probability of a test reporting a significant effect. By contrast, the probability of a significant effect increases when the relationship was subject to a limitation (sex-specific, time-specific, interactions with body mass, etc.) by 2.5–9.7% (50% cred. int; post. mean = 8.87, 95% cred. int. = 0.57–40.25 *P*(*β*_Limitation_) < 0 = 0.004).

Tests of specific entities have a 0.14–0.37 (50% cred. int.; post. mean = −0.26, 95% cred. int. = −0.64 to 0.002) lower probability of reporting a significant effect than those of broad entities (specific: post. mean = 0.91, 95% cred. int. = 0.02–0.25; broad: post. mean = 0.34, 95% cred. int. = 0.08–0.71, *P*(*β*_Broad_ > *β*_Specific_) = 0.973; figure 2a). There was no evidence of differences in the probability of returning significant effect in tests across other cognitive levels (*P*(*β*_Broad_ > *β*_General_) = 0.803, *P*(*β*_General_ > *β*_Broad_) = 0.760; figure 2a).

Tests using survival as the (proxy) fitness measure have a 0.22–0.44 (50% cred. int; post. mean = 0.33, 95% cred. int. = 0.04–0.67) greater probability of reporting a significant effect than tests using reproduction as a fitness measure (survival: post. mean = 0.43, 95% cred. int. = 0.18–0.73; reproduction: post. mean = 0.09, 95% cred. int. = 0.02–0.25, *P*(*β*_Survival_ > *β*_Reproduction_) = 0.985; figure 2c). We found only weak support for the hypothesis that different cognitive levels vary in their relationship with different proxy fitness measures (*Δ*^F^): for most contrasts the difference in probability of finding a significant effect across the two fitness measures is similar for different cognitive levels (*P*(*Δ*^F^_Specific_ > *Δ*^F^_Broad_) = 0.282, *P*(*Δ*^F^_General_ > *Δ*^F^_Broad_) = 0.761, *P*(*Δ*^F^_General_> *Δ*^F^_Specific_) = 0.885; figure 2d). However, there is an 88.5% probability that the difference in probability of finding a significant effect between the two fitness measures is larger in tests of general entities than specific entities.

### (c) Are there differences in patterns of relationships depending on whether the relationships are positive or negative?

**Figure 3.**
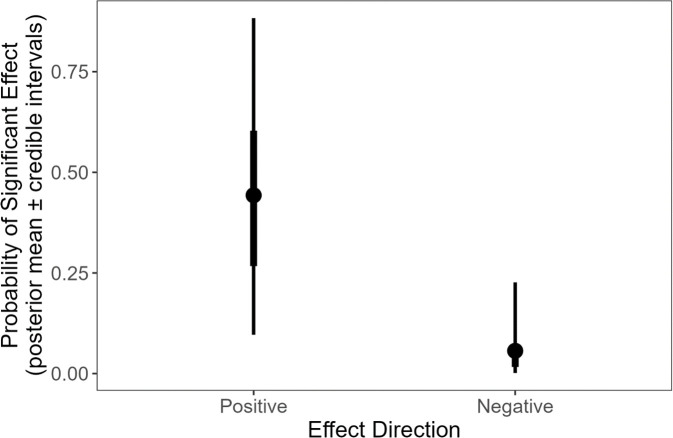
Predicted probability of a test reporting a significant positive or negative effect, derived from the posterior of the fitted Effect Direction model. Points show the posterior means, thicker error bars show the 50% credible intervals and the thinner error bars the 95% credible intervals.

Overall, the probability of a significant reported effect being positive is 52–92% (50% cred. int.; post. mean = 0.53, 95% cred. int. = −1.64 to 0.994) higher than the reported effect being negative (*P*(*β*_Positive_ > *β*_Negative_) = *Δ*^+/−^ = 0.985) (figure 3). This pattern is consistent across cognitive levels being tested: the probability of a significant result being positive compared with negative does not differ across the cognitive levels being considered (*P*(*Δ*^+/−^_Broad_ > *Δ*^+/−^_Specific_) = 0.737, *P*(*Δ*^+/−^_Broad_ > *Δ*^+/−^_General_) = 0.505, *P*(*Δ*^+/−^_General_ > *Δ*^+/−^_Specific_) = 0.737). The pattern is also consistent across fitness measures: the probability of a test reporting a significant positive compared with a negative effect does not differ between survival and reproduction fitness proxies (*P*(*Δ*^+/−^_Survival_ > *Δ*^+/−^_Reproduction_) = *Δ*^+/−, F^ = 0.606). This pattern also extends to within cognitive levels: we find no evidence that the difference in the probability of finding a positive versus a negative effect in tests with survival compared with reproductive fitness measures differs between cognitive levels (*P*(*Δ*^+/−, F^_General_ > *Δ*^+/−, F^_Broad_) = 0.302, *P*(*Δ*^+/−, F^_Broad_ >*Δ*^+/−, F^_Specific_) = 0.433, *P*(*Δ*^+/−, F^_General_ > *Δ*^+/−, F^_Specific_) = 0.315).

## 4. Discussion

There is now a body of 45 studies containing 211 relationships linking aspects of an individual’s cognitive performance in a range of cognitive entities to their fitness, and thus indicating how selection may operate to drive the evolution of cognition. Our analysis of these studies revealed several key patterns in the relationship between cognitive performance and fitness outcomes. The majority of relationships (71%) were neutral, with positive relationships (‘smarter is better’) outnumbering negative ones (‘dumber is better’) among the significant results. The probability of reporting a significant effect varied with sample size, cognitive level and the fitness measure used. Specifically, survival fitness measures were more likely to show significant relationships than reproductive fitness measures. Additionally, we observed that the likelihood of positive relationships outweighed negative ones, a pattern consistent across cognitive levels and fitness measures. These studies cover a wide range of taxa, although there is a strong bias towards vertebrates, especially birds. The studies consider a wide variety of definitions of cognition, cognitive entities and cognitive levels. The majority focus on single, specific entities, but others consider measures of broader entity, with studies involving multiple tasks providing a measure of general (cognitive) entity unsurprisingly being least common. They also consider a variety of potential links to fitness, with most focusing on fitness accruing through reproductive success rather than survival. Most of these studies were conducted on free-living animals (although some captured individuals for testing periods, e.g. [41,44], others reared and tested individuals in captivity before release to the wild to measure fitness, e.g. [26,45], some others were entirely laboratory-based, e.g. [20,42], so a range of selective forces could operate naturally, giving ecological validity to the work).

Overall, this body of work currently provides rather weak support for the idea that an individual’s cognitive performance as measured by psychometric tasks is consistently related to their (proxy) fitness. We suspect that even this might be an overestimate of the actual proportion, given that non-significant results are, or historically have been, harder to have accepted for publication [46]. Furthermore, the likelihood of a significant relationship being detected within those published results declines as the sample size, and presumably power increases. This supports the idea that published results might be unrepresentative [47]. In addition, of those relationships that were significant, almost 70% were only likely to apply to particular categories of individuals or under particular environmental conditions, meaning that consistent selection at a species level would be difficult to maintain. Consequently, studies that included some form of limitation had a higher probability of reporting a significant relationship. This may be explained in three ways. First, authors may search their data after collection to uncover significant, albeit limited, relationships that support publication. In doing so, the presence of one or more ‘limitations’ represents a bias [48]. Second, authors might be deliberately and *a priori* restricting their analyses to classes of individuals or specific ecological scenarios that they believe are especially likely to gain fitness benefits from cognition. It is notable that none of the studies that we included was pre-registered, which might allow us to discount these two explanations. Finally, cognition may only relate to fitness under very particular, limited circumstances, and consequently selection is expected to be sporadic or inconsistent across the species, meaning that the long-term strength and direction of evolution is difficult to predict.

Where significant relationships were reported, almost 80% of raw relationships indicated that exaggerated expression of cognitive performance (faster learning; more accurate memory; stronger executive control) was positively related to (proxy) fitness outcomes. It was even more likely to find a positive relationship once potential influential co-variates have been controlled for. Such exaggerated performances might be considered to be the mark of a smarter individual. Typically, when studying the evolution of cognition, it is this exaggeration of cognitive performances (and correspondingly larger brain size, e.g. [49], but see [50]) that is of interest. Such exaggeration is expected to provoke costs (increased energy demands, e.g. [22]), and findings that these provide fitness gains help to explain the benefits. In general, therefore, we can conclude that when selection acts on cognition, it tends to exaggerate performances of cognitive entities. However, a notable proportion of relationships were in the opposite direction (23% of the raw relationships and a 7% likelihood of drawing a relationship once potential influential co-variates have been controlled for), with slower learning, less accurate memory, and weaker executive control being related to fitness benefits. From our analyses, it is not possible to determine whether this is because of energetic or other costs of the entity, trade-offs with other cognitive entities or performances, or because there are behavioural benefits from, e.g. taking longer to acquire new information or showing lower cognitive flexibility.

Significant relationships were more likely if the fitness measure considered corresponded to the survival of an individual rather than its reproductive output. This is despite most studies focusing on reproductive output, which should provide a more direct measure of contributions to future generations, and for which the role of cognition in reproduction via sexual selection has been well discussed, with a particular focus on mate choice [51] and contests [52]. In the studies that we reviewed, none of the (proxy) fitness measures related to reproduction directly represented total contributions to future generations (electronic supplementary material, S2). Instead, the closest measures were of numbers of young surviving to (apparent) independence from a single year’s reproductive output [15,18,41,53–57]. However, many studied species are long-lived (species list in electronic supplementary material, S2). Some take several years before reaching reproductive maturity, and all typically have multiple breeding seasons, so the output of a single year may be poorly related to lifetime reproductive success. This weak link might explain why significant relationships were less likely. To progress our understanding of how reproductive output might depend on cognition, more studies of short-lived or semelparous species are desirable. Indeed, we suspect that different cognitive entities will play critical roles for short- versuss. long-lived species.

It is perhaps surprising that it was more likely that relationships with survival were found. Rochais *et al.* [52] describe a series of methodological and theoretical impediments to linking cognition to survival, noting that the survival of an individual depends on a disparate range of short-term and long-term factors, including foraging ability, disease resistance, competitive ability, and predator detection and avoidance. Consequently, there are many ways that cognition could benefit an individual. This might explain why the pattern (that relationships with survival were more likely) was especially strong when considering relationships at the level of general entities as compared with broad entities. To better understand how selection acts on cognition via survival, it may be helpful for future studies to first determine and define how much an individual’s survival for different lengths and at different stages determines their contributions to future generations and to focus on mechanistic links where particular factors, with an *a priori* defined cognitive dependence, are especially influential in affecting survival.

A significant relationship with a fitness measure (of either type) was also more likely if the cognitive level involved broad as opposed to specific entities. We found this surprising because we expected that researchers might deliberately choose to study specific entities with clear *a priori* links to fitness consequences postulated or identified and so be more likely to detect relationships with such entities. We suggest two possible explanations for this. First, assays of broad entities might inherently capture a wide range of specific entities that all contribute to the individual’s performance in the problem-solving or innovation task [58]. Consequently, there is a greater chance that any one of these might be related to the fitness measure and thus a relationship might be detected. Second, it may be that behaviours are more dependent on more broad or general entities and selection acts on the whole or at a higher level, rather than the component-specific entities.

The weak and rather inconsistent link between cognitive performance and fitness measures may arise because the cognitive entity of interest is not highly specific to just the one behaviour measured but contributes to a suite of different behaviours and is sensitive to a range of stimuli in different contexts. For example, spatial memory might be used to recall foraging patches, landscapes of fear, territories of attractive mates or the location of rewards in a psychometric test. This assumption underpins the rationale that cognitive entities can be assayed using psychometric tests, independent of the natural behaviour that they are believed to influence, and which is traditionally considered to be the focus of selection (but see [9]). This assumption has two consequences, both likely to weaken detectable relationships with fitness. First, the disconnect between what is being measured (the test performance) and what is being selected (the behaviour) complicates any relationship. Second, this abstraction, separating the test from specific real-world behaviour, implies (and is perhaps motivated by the assumption) that the cognitive entity of interest might contribute to more than one behaviour. This is exciting because it means that selection acting on one behaviour that is shaped by a cognitive entity might, through changes it exerts on that entity used in one context, also affect the expression of other behaviours that are underpinned by the same cognitive entity. However, it also means that focusing on specific proxy fitness measures might provide an incomplete description of how the cognitive entity affects multiple aspects of the individual’s behaviour and alternative fitness consequences. To progress the field, it would be helpful to better understand how cognitive entities contribute to multiple behaviours and how selection on any one of these behaviours might feed back to alter cognitive entities that in turn alter other behaviours and their fitness consequences. The current body of studies is primarily interested in bivariate relationships, with those measuring multiple entities or behaviours paying little attention to the links between them.

This novel perspective on how selection might operate, causing correlated changes in multiple behaviours, analogous to pleiotropy, with attendant negative consequences or trade-offs across different behaviours, offers a richer understanding of the evolution of cognition. However, it also raises questions about how cognitive entities might operate, relate to one another and relate to different behaviours. Much may depend on whether there is any structure linking different cognitive entities to one another and whether or how information flows between them to facilitate decision-making. There is no agreed *a priori* structure of Cognition [2,59]. One suggested model proposes that behaviour results from a strict chaining of cognitive entities that directly and invariably feed one another in a bottom-up conceptualization [59]. If a linear structure operates then we might expect that selection on the behaviour then feeds back down the same route, and we might expect that selection acts first on the terminal entity (typically decision-making) and only when this is optimized/fixed can selection start to act on the preceding entities (perhaps aspects of perception, learning and/or memory). An alternative suggested model structure posits that information is processed in parallel or there may be feedback loops (e.g. attention is modified [2]), in which case selection might be expected to act on any part of the structure independently and each entity can be adapted independently of the others. Such different conceptions of the structure and specificity of cognition might have very different consequences for selection and thus how we might hypothesize relationships between fitness and cognition. Currently, researchers make assumptions about exactly what cognitive entities are involved and thus examined by their studies, how they interact with one another and how they may determine either the natural behaviour that is likely subject to selection pressures or how they determine an individual’s performance in the psychometric test deployed to assess them. These assumptions are usually implicit and seldom described. However, it is vital to consider them in detail because the assumptions have major implications for (i) devising and administering psychometric tests, (ii) predicting and interpreting outcomes from those tests, and (iii) understanding evolutionary changes and constraints. Currently, none of the studies we reviewed specifies how it envisages the structural relationship within and between cognitive entities and the behaviours that they underpin, yet this is critical to make predictions as to how selection might operate, and hence the evolutionary consequences.

## 5. Conclusions

Our understanding of the evolution of cognition, taking a Cognitive Ecology approach, remains incomplete. Despite compelling individual relationships, the overall body of existing studies provides weak evidence for a consistent link between cognition and (proxy) fitness outcomes, making it difficult to envision how selection might consistently drive cognitive evolution. However, the studies suggest that fitness consequences of cognition may be stronger or more common when considering survival of individuals rather than reproductive output, and when broader or more general cognitive entities are considered. This indicates that behavioural outcomes may result from the interplay of multiple cognitive entities. The Cognitive Ecology approach, which links performance in abstract tasks to fitness outcomes, offers the potential to explore interactions between different cognitive entities, their trade-offs, performance constraints and the fine-scale links to behaviours. This perspective opens up novel avenues for understanding the evolution of cognition, complementing the comparative approach by considering how selection might act on multiple cognitive systems in complex, dynamic environments.

## Terminology used in this article

**Figure 4.**
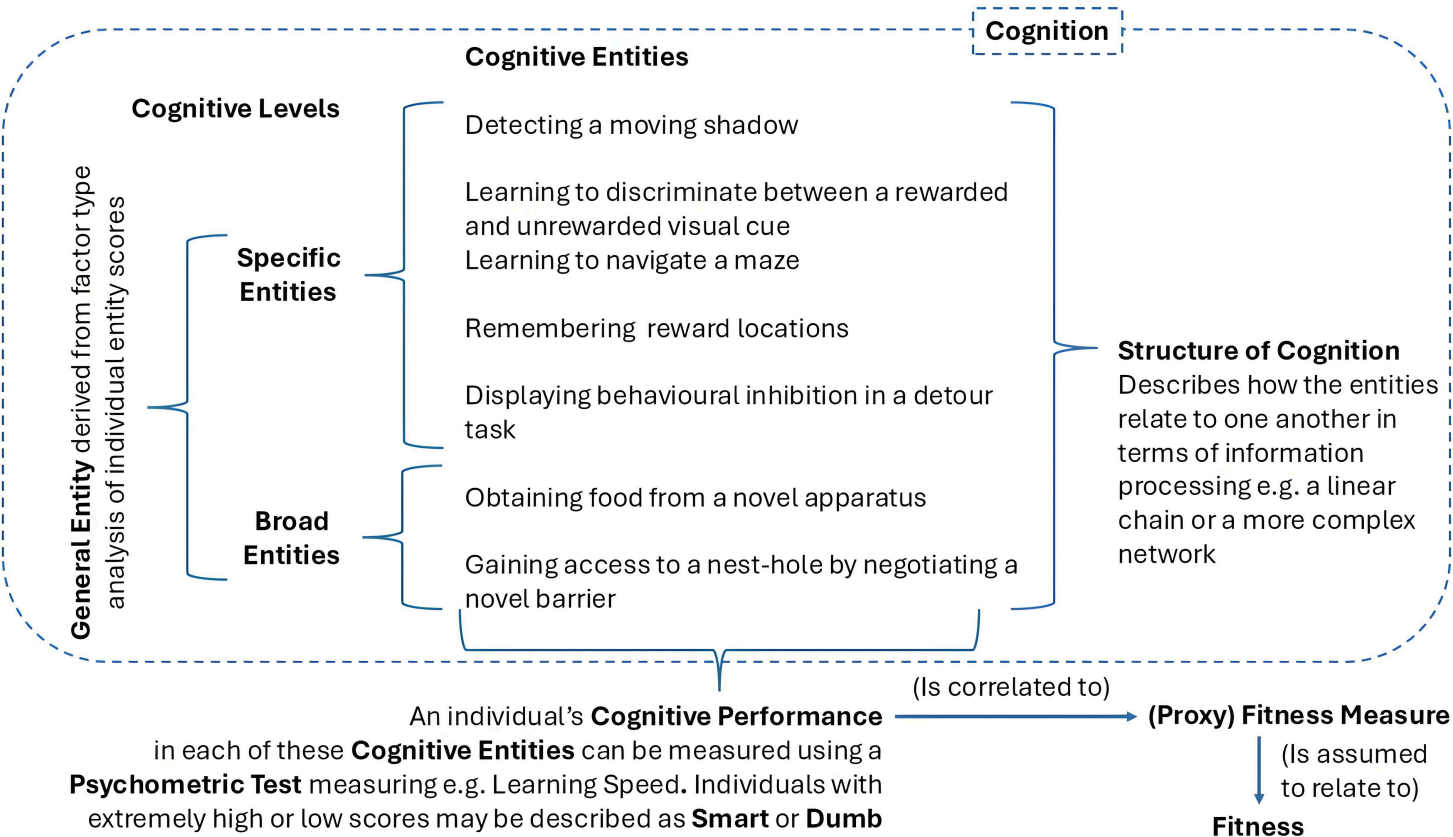
Depiction of how the terms used in this paper referring to cognition relate to one another. Terms in bold are defined in the Glossary at the end of the article.

The field of Animal Cognition contains many terms that are interpreted differently by different researchers and which are applied in different ways. This becomes especially problematic when conducting a systematic review, where the same terms are deployed to refer to different concepts, measures or entities. We have provided a Glossary and figure 4, where we explain and illustrate what we mean when using particular ambiguous or contentious terms and briefly outline how they have been used by others in the field. We capitalize and underline a term that we define in the Glossary the first time it is used in the main text.

## Glossary

**Table:** 

**Cognition—**In this article, we adopt the definition of Shettleworth [60], with Cognition referring very broadly to the neural processing of information from the environment, including the acquisition, storage, retrieval and use of that information for making behavioural decisions. When we talk about the evolution of cognition, this is a shorthand encapsulating both the origins of Cognitive Entities and exaggeration of Cognitive Performances. **Cognitive Entity**—We use this term in preference to the more commonly used Cognitive Ability (e.g. [35,49,61–64]) or Cognitive Measure (e.g. [65]) or the use of the term Cognition as a shorthand (e.g. [66,67]). We do so because it explicitly differentiates between two very different uses of the term Cognitive Ability which can cause confusion. Cognitive Ability can refer to the performance of an individual in a task or behaviour deemed to depend on cognition (e.g. [61,62]), such that individuals vary in the level of performance the exhibit, with selection assumed to act on this variation to exaggerate it. This is what we call Cognitive Performance. Cognitive Ability may also serve to describe some discrete cognitive unit, for example the ability to solve a problem [18,41], with individuals varying whether the ability is present or not, with selection favouring the presence (or absence) of that ability. We call such discrete units Cognitive Entities. We accept that neurally, and probably cognitively, such Entities are not truly discrete, likely involving a network of neurons. However, they are certainly treated as such by researchers in this field, who typically conduct tests ‘for’ particular (discrete) Cognitive Entities (‘We studied spatial memory’, ‘We measured inhibitory control’), with little discussion about the correlations between putative Entities. Therefore, we follow this approach in our definition here. **Cognitive Performance**—The measure that an individual achieves in a Psychometric Test (e.g. [15,44,55–57,68–71]). This performance is typically assigned a qualitative value by the researcher, for example, a test individual that learns to discriminate between two cues after fewer trials is deemed to be a fast learner. Researchers in Cognitive Ecology generally envisage that Cognitive Performance is under selection, and structure their analyses to link Cognitive Performance with a (proxy) Fitness Measure of interest. (**Abstract) Psychometric Tests**—Tasks that allow researchers to quantitatively differentiate between individuals in their Cognitive Performance (*q.v.*) in a particular Cognitive Entity (*q.v.*). This may be a binary distinction (pass/fail) suggesting presence or absence of a Cognitive Entity (e.g. an individual can or cannot solve a problem [18,41,72]), or a more continuous measure indicating the strength of the Cognitive Performance (e.g. individuals can be ordered by the speed at which they learn a discrimination or reward locations [45,57]). Such tests may be described as Abstract when they use cues or arrangements that are not naturally encountered (e.g. coloured discs [15,26], or evenly spaced circular arrangements of reward locations [13,54]), but which are assumed to call upon the same Cognitive Entities as natural behaviours that are subject to selection, owing to the assumed generality of cognitive entities across behaviours. **Smarter/Dumber**—These describe an individual exhibiting a high (Smarter—e.g. learning an association faster, making fewer errors in a spatial task, exhibiting stronger or more rapid behavioural inhibition) or low (Dumber—e.g. learning an association slower, making more errors in a spatial task, exhibiting weaker or slower behavioural inhibition) Cognitive Performance score. Although the applicability of such terms is open to debate and may be value-laden, distorting the fact that selection will favour a Cognitive Performance that is better fitted to its socio-ecological background, which may involve learning slower, forgetting more or making more errors, in this article we use them, as do others, e.g. [43], as an easy-to-understand shorthand to describe individuals with Cognitive Performances that are exaggerated in these dimensions. **Cognitive Level**—This is a term that we use for convenience in this article. It provides a crude and subjective method of describing how specific the Cognitive Entity being studied is. We have assigned entities to one of three levels of decreasing specificity: Specific Entities, Broad Entities and General Entities. **Specific Entities**—These comprise Cognitive Entities that are treated as a discrete unit by the researchers; e.g. learning to discriminate between a rewarded and unrewarded visual cue [69], detecting a moving shadow [16] or remembering reward locations [45]. These entities are often explicitly associated with one or a few very similar behaviours, and so are thought to be selected for by a small number of natural behaviours. **Broad Entities**—A Cognitive Entity with a specific name and treated as a discrete unit by the researchers but likely to involve a range of constituent but unspecified Specific Entities, testable with a single assay, e.g. ‘problem solving’, ‘flexibility’, ‘innovation’. Such Entities may lack a demonstrable underlying psychology or neuroscience that links the network of Specific Entities, but they are commonly conceived and referred to by researchers in this field as being discrete and identifiable. Such Broad Entities are often considered to be applicable in a wide range of natural situations and so likely subject to selection through multiple different behaviours. **General Entities**—A construct of the researchers, being some form of amalgamated score derived from a series of Cognitive Performances collected across a suite of Cognitive Entities (maximum of seven in the studies in this article [20]) combined to produce an index, analogous to ‘*g*’ (e.g. [15,17]). Such General Entities are considered to capture some overall measure of an individual’s Cognition and thus are expected to influence all behaviours that involve Cognition. **Structure of Cognition**—The way by which information is acquired and processed leading to behaviours. Conceptions of the Structure of Cognition are diverse and often poorly specified [2,59,60]. Crudely, it may be considered analogous to neural structure, with links between particular neurons, or groups of neurons in networks, mapping onto links between Cognitive Entities. At one extreme, there may be no structure, with every Cognitive Entity operating entirely independently of all others. Alternatively, Entities might be arranged in some form of chain, where information is acquired through perceptual Entities, learned and remembered and then used while decision-making. Finally, the structure may be more complex, with a network of connections between Entities allowing feedback and fine-tuning between them. **Fitness**—Commonly considered to be a product of an individual’s contribution to future generations, mediated by its survival [73]. For example, to determine an individual’s lifetime reproductive output would require that the individual be followed in a natural setting until its death and counting its reproductive output (including any extra-pair parentage and/or indirect reproductive success accruing as a result of helping relatives) be recorded. **Proxy Fitness Measures**—Measures either proven or believed to relate to ‘true fitness’, albeit imperfectly, but which can be obtained over much shorter timescales covering only a portion of an individual’s lifespan (e.g. the reproductive output of an individual for one year [57]; or the number of mates that it can attract [17,62]), or which rely on behaviours directly or indirectly linked to survival (e.g. the efficiency of foraging success [67,74]; mass changes [75]; or probability of winning an intra-specific contest [70]).

## Data Availability

All data and code are included as electronic supplementary material available online [76].
